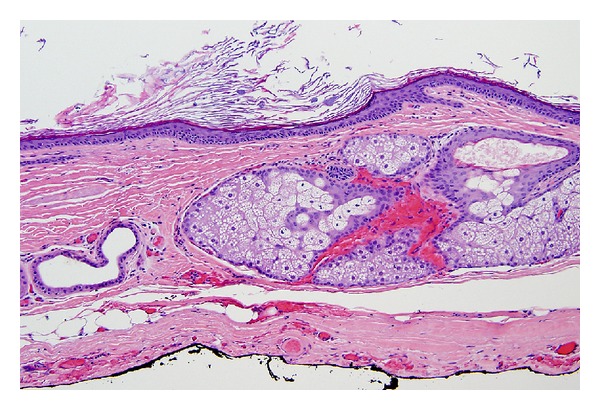# Primary Teratoma of the Lesser Sac: Lesser Sac Teratoma

**DOI:** 10.1155/2012/604571

**Published:** 2012-03-26

**Authors:** Brandon M. Hardesty, Thomas M. Ulbright, Christopher Touloukian, Lawrence H. Einhorn

**Affiliations:** Indiana University Simon Cancer Center, 535 Barnhill Drive, Indianapolis, IN 46202, USA

## Abstract

Germ cell tumors predominantly involve the gonads but may rarely be found outside of the gonads, primarily in midline structures. We describe the case of a 27-year-old male with an asymptomatic 8 cm teratoma located within the lesser sac of his omentum. This is the fourth case of a teratoma located within the lesser sac of the omentum, which provides the opportunity to make some comparisons. Finally, we discuss some of the etiologic theories behind extragonadal germ cell tumors and how they relate to teratomas in the lesser sac.

## 1. Introduction

Extragonadal germ cell tumors are well described in the literature and comprise 1–5% of all germ cell tumors. Primary abdominal or retroperitoneal germ cell tumors comprise approximately 5% of these extragonadal tumors [1]. Among adults, germ cell tumors demonstrate a marked male predominance and are almost exclusively restricted to midline structures [2]. There have been several reports of tumors arising from the stomach [3–5], mesentery [6], and pancreas [5], but only three other cases of adults with teratoma originating in the lesser sac of the omentum [7–9], all of which were contained within the hepatoduodenal ligament. We present the fourth description of an adult with a mature teratoma located in the lesser sac of the omentum, the first originating from the left side of the celiac axis.

## 2. Case Presentation

**Figure 1:**
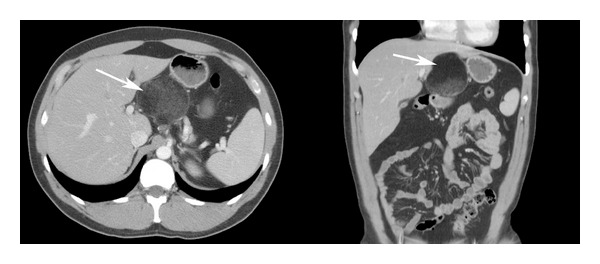


A 27-year-old man without past medical history presented with acute appendicitis, which was treated with laparoscopic appendectomy. During evaluation, he was found to have a large (5 × 5 cm) fibro-fatty mass in his lesser sac, adjacent to the celiac plexus (Figure 1). He was completely asymptomatic from the mass. The initial differential diagnosis based on CT scan was benign lipoma or possibly low-grade liposarcoma. The patient subsequently underwent surgical resection, which revealed a mass emanating from the lesser sac. The common hepatic artery was not affected, but the left gastric artery was involved by this tumor. No lymphadenopathy was observed. Pathologic examination revealed an 8.5 × 7.9 × 7.0 cm cystic structure, which on sectioning demonstrated grumose material with a moderate amount of hair. No teeth or calcified structures were identified. On microscopic examination, the cyst was lined by stratified squamous epithelium with associated hair follicles, sebaceous glands, and sweat glands (Figure 2). The final pathologic diagnosis was mature cystic teratoma (dermoid cyst). There was no evidence of a malignant germ cell component. Subsequent testicular ultrasound demonstrated no evidence of a testicular primary. Beta-human chorionic gonadotropin and alpha-fetoprotein were checked postoperatively and were within normal limits. The patient was seen 4 months postoperatively with a normal abdominal CT scan.

## 3. Discussion

Gastrointestinal teratomas are rare tumors, which predominantly occur in children [10], presumably related to either abnormal migration of the primordial germ cells during embryogenesis or ectopic pluripotential cells [2]. We describe not only the fourth case of teratoma within the lesser sac of the omentum in an adult, but the first case that appears to arise from the left side of the celiac axis. The three prior adult cases of teratoma within the lesser sac appeared to arise from the hepatoduodenal ligament [7–9]. Two of the prior cases were women. In an examination of the four reported cases of teratoma within the lesser sac, some common features emerge. All patients, with the exception of ours, presented with abdominal pain and on subsequent evaluation were found to have epigastric masses ranging from 6 to 11 cm. Three presented in their late twenties with the fourth presenting at 38 years of age. All had complete resection of their tumors and had no evidence of disease recurrence on followup as long as 33 months.

 The precise etiology for these tumors has not been elucidated. The most widely accepted theory is that they derive from primordial germ cells that experience aberrant migration and subsequently fail to undergo apoptosis. Perhaps arguing against this theory is the fact that there are no cases of germ cell tumor, apart from teratoma, that originate in the lesser sac of the omentum. Alternatively it has been proposed that these tumors are formed by pluripotent embryonic stem cells that escape normal differentiation and later develop into teratomas, although this phenomenon predominantly occurs in infants [2]. Our case and the prior ones may conceivably represent extremely late presentations of the same disease process that occurs in infants, who develop teratomas in similar locations [10, 11]. However, one would have expected these patients to have developed symptomatic growth of their teratoma much earlier than the third decade of life. A third theory that has been hypothesized for extragonadal germ cell tumors is the dedifferentiation of already established malignancies (so-called “neometaplasia”), which has been proposed in cases having both a germ cell tumor and a more common malignancy such as gastric adenocarcinoma [4, 5]. In our case as well as the other adults with lesser sac teratomas, there was no evidence of another concurrent malignancy, suggesting that the teratomas arose independently of other malignant processes. The young age of the patients is an additional argument against this pathogenesis.

 In summary, we present the first case of an extragonadal teratoma occurring primarily on the left side of the celiac axis. This is also the fourth case of an extragonadal teratoma with an origin within the lesser sac of the omentum. These extremely rare tumors should be kept in the differential diagnosis of a mass originating within the lesser omentum as their prognosis appears to be excellent following complete surgical resection.

## Figures and Tables

**Figure 2:**